# The Highly Conserved Barley Powdery Mildew Effector *BEC1019* Confers Susceptibility to Biotrophic and Necrotrophic Pathogens in Wheat

**DOI:** 10.3390/ijms20184376

**Published:** 2019-09-06

**Authors:** Yi Zhang, Kedong Xu, Deshui Yu, Zhihui Liu, Chunfeng Peng, Xiaoli Li, Ju Zhang, Yinghui Dong, Yazhen Zhang, Pan Tian, Tiancai Guo, Chengwei Li

**Affiliations:** 1 Key Laboratory of Plant Genetics and Molecular Breeding, Zhoukou Normal University, Zhoukou 466001, China (Y.Z.) (K.X.) (D.Y.) (Z.L.) (C.P.) (X.L.) (J.Z.) (Y.D.) (Y.Z.) (P.T.); 2 Henan Engineering Research Center of Grain Crop Genome Editing, Henan Institute of Science and Technology, Xinxiang 453003, China; 3 The Collaborative Innovation Center of Henan Food Crops, Agronomy College, Henan Agricultural University, Zhengzhou 450002, China; 4 Henan Key Laboratory of Crop Molecular Breeding & Bioreactor, Zhoukou 466001, China

**Keywords:** effector protein, *Blumeria graminis* f. sp. *tritici*, *Gaeumannomyces graminis* var. *tritici*, haustorium formation, necrosis development, cell death

## Abstract

Effector proteins secreted by plant pathogens play important roles in promoting colonization. *Blumeria* effector candidate (*BEC*) 1019, a highly conserved metalloprotease of *Blumeria graminis* f. sp. *hordei* (*Bgh*), is essential for fungal haustorium formation, and silencing *BEC1019* significantly reduces *Bgh* virulence. In this study, we found that *BEC1019* homologs in *B. graminis* f. sp. *tritici* (*Bgt*) and *Gaeumannomyces graminis* var. *tritici* (*Ggt*) have complete sequence identity with those in *Bgh*, prompting us to investigate their functions. Transcript levels of *BEC1019* were abundantly induced concomitant with haustorium formation in *Bgt* and necrosis development in *Ggt*-infected plants. *BEC1019* overexpression considerably increased wheat susceptibility to *Bgt* and *Ggt*, whereas silencing this gene using host-induced gene silencing significantly enhanced wheat resistance to *Bgt* and *Ggt*, which was associated with hydrogen peroxide accumulation, cell death, and pathogenesis-related gene expression. Additionally, we found that the full and partial sequences of *BEC1019* can trigger cell death in *Nicotiana benthamiana* leaves. These results indicate that *Bgt* and *Ggt* can utilize *BEC1019* as a virulence effector to promote plant colonization, and thus these genes represent promising new targets in breeding wheat cultivars with broad-spectrum resistance.

## 1. Introduction

Wheat makes a substantial contribution to human calorie intake worldwide (Food and Agriculture Organization of the United Nations, http://www.fao.org/faostat/en), and plays a major role in ensuring global agricultural sustainability and food security [1]. The recent release of a high-quality, fully annotated and ordered bread wheat genome offers considerable promise for future developments in wheat cultivation [2]. However, recently published data indicate that global losses in wheat yield attributable to pathogenic fungi and pests account for approximately 21.5% of total losses, reaching up to 28.1% in food-deficit areas [3]. Although the planting of resistant cultivars and application of various fungicides have been the primary approaches adopted by farmers to stem the increasing incidence of plant fungal diseases, the latter is a potential source of environmental pollution [4]. Moreover, current agricultural practices typically involve the planting of single-genotype cereal crops over extensive areas, accelerating the selection of fungal strains that can overcome inherent crop genetic resistance [4]. There is, accordingly, a constant demand for innovative developments in sustainable agricultural and the introduction of novel resistance strategies in cereal crop breeding [4].

Based on their life histories, plant pathogens can be divided into those that kill the host and absorb nutrients from dead cells (necrotrophs) and those that require a living host to complete their life cycle (biotrophs) and those that have an initial biotrophic phase, then become necrotrophic (hemi-biotrophs) [5] and employ diverse strategies relating to host interaction. Plant defense responses consist of two layers designed to resist pathogen attack, namely, pathogen-associated molecular pattern (PAMP)-triggered immunity (PTI) and effector-triggered immunity (ETI) [6,7]. PAMPs, such as flagellin, chitin, and EF-Tu, are perceived by plant pattern recognition receptors (PRRs), thereby initiating a series of cellular signals, including the activation of a downstream mitogen-activated protein kinase cascade and induction of defense-related genes [6,8,9]. ETI, the second layer of plant defense, is activated by pathogen effectors, secreted by fungi, modulating host plant physiology to accommodate the fungi and provide the necessary nutrients [10,11]. Effectors are mostly recognized by nucleotide-binding site and leucine-rich repeat (NBS-LRR) proteins, encoded by host resistance (*R*) genes, involved in inhibiting pathogen colonization [10,11]. The direct and indirect interactions between effectors and R proteins lead to resistance, often accompanied by a hypersensitive response (HR) and local cell death that prevent pathogen colonization [8,12].

Take-all disease, caused by the soil-borne necrotrophic fungus *Gaeumannomyces graminis* var. *tritici* (*Ggt*), is an important root disease of cereals, capable of reducing grain quality and grain yield by 40–60% [13,14]. Upon infection, *Ggt* hyphae penetrate the root cortical cells and progress upward into the base of the stem, wherein they extract nutrients from the plant, causing premature death. The symptoms manifest as chocolate brown to black lesions on the infected roots and stem bases, premature ripening, and whiteheads [15]. Although breeding resistant wheat cultivars is considered the most promising and reliable measure to protect wheat from take-all, no effective resistant wheat varieties have been identified to date and traditional crop rotation is neither economically feasible nor practicable [16]. Recently, the use of beneficial microorganisms (e.g., *Pseudomonas fluorescens*, *Bacillus subtilis*, *Bacillus velezensis,* and *Serratia proteamaculans*) has been identified as an alternative, effective, and eco-friendly strategy for the control of take-all disease [17,18,19,20].

*Blumeria graminis* f. sp. *tritici* (*Bgt*), the causative agent of wheat powdery mildew, is an obligate biotrophic fungus and a major threat to common wheat (*Triticum aestivum* L.) production worldwide [21]. In a compatible interaction between wheat and *Bgt*, the fungal conidia germinate and penetrate the plant epidermal cell walls, and thereafter acquire nutrients by forming a specialized feeding structure, the haustorium, which is crucial for the subsistence of these obligate biotrophs [12,21]. Subsequently, a series of effector proteins are secreted into plant cells by the pathogen, which manipulate plant defense mechanisms and serve to promote colonization [22,23]. The interaction between barley and powdery mildew *B. graminis* f. sp. *hordei* (*Bgh*) is considered an ideal pathosystem in monocots for examining the influence of effectors on host genetic and molecular mechanisms [24,25]. Four avirulence (Avr) proteins AVRa1, AVRa13, AVRK1, and AVRA10, identified in *Bgh* are respectively recognized by the barley resistance proteins MLA1, MLA13, MLA10, and MLK1, which trigger HR in the host [24,25,26].

Proteomic and genomic studies have identified more than 500 *Blumeria* effector candidates (*BECs*) or candidate secreted effector proteins (*CSEPs*), for which homologous genes have been identified in *Bgh* and *Bgt* [27,28]. Although only a few *BECs* and *CSEPs* have been characterized, they have been shown to play vital roles in virulence. For example, the secreted *Bgh* effectors *CSEP0105* and *CSEP0162* significantly reduce the rate of fungal haustorium formation and interact with the heat shock proteins Hsp16.9 and Hsp17.5 [29]. Additionally, host-induced gene silencing (HIGS) of *CSEP0055*, which interacts with barley pathogenesis-related protein PR17c, reduces fungal aggressiveness [30]. Similarly, the ribonuclease-like effector candidate *BEC1011* has been found to act as a suppressor of pathogen-induced host cell death [31]. Previous research has revealed that certain effectors are vital for haustorium development [32], and silencing of the *Bgh* effector *BEC1019*, which encodes a secreted metalloprotease, significantly reduces *Bgh* virulence in barley [33,34]. Additionally, *BEC1019* is broadly conserved across a diverse range of organisms, including 37 plant pathogens (comprising major pathogens of rice, maize, and wheat), 15 animal pathogens, 4 insect pathogens, and 35 non-pathogens [33,34]. These observations, therefore, indicate that the conserved effector gene *BEC1019* could potentially serve as a broad-spectrum target for the control of different pathogens.

The objectives of the present study were three-fold, namely, to (i) identify and align homologous genes in eight plant pathogens (including biotrophs, hemi-biotrophs, and necrotrophs) with the *BEC1019* gene in *Bgh*, (ii) overexpress and silence *BEC1019* in wheat to investigate its roles in the colonization of *Bgt* and *Ggt*, and (iii) determine whether hydrogen peroxide (H_2_O_2_) accumulation and cell death are involved in wheat disease resistance.

## 2. Results

### 2.1. BEC1019 is Broadly Conserved in Plant Fungal Pathogens

**Figure 1:**
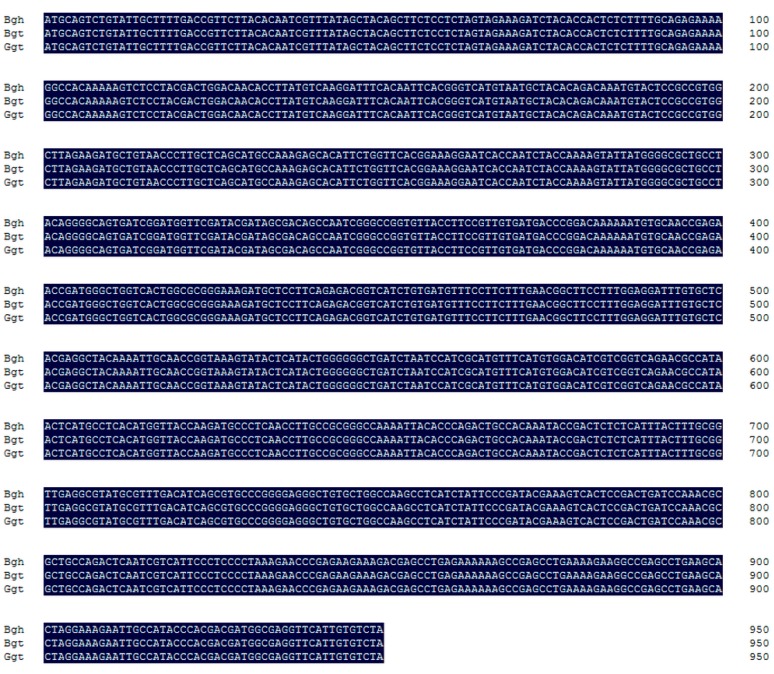
Nucleotide sequences of the homologous genes in *Blumeria graminis* f. sp. *tritici* (*Bgt*) and *Gaeumannomyces graminis* var. *tritici* (*Ggt*) were completely identical to the *BEC1019* sequence in *B. graminis* f. sp. *hordei* (*Bgh*).

*Bgt* and *Bgh* are the two main formae speciales of *B. graminis*, and genome-wide analyses have indicated that almost 92% of the genes in *Bgt* and *Bgh* are homologous [27,28]. *BEC1019* has been demonstrated to play key roles in the development of resistance to *Bgh* in barley [33,34]. Accordingly, we searched for a gene in *Bgt* homologous to the *BEC1019* gene of *Bgh*. To identify the *BEC1019* homolog in *Bgt*, primers designed based on *Bgh BEC1019* and *Bgt* DNA were used to clone the candidate gene. The sequencing results showed that the nucleotide and protein sequences (without signal peptides) of *Bgt BEC1019* are completely identical to those of *Bgh BEC1019* (Figure 1). To analyze the conservation of *BEC1019* in the diverse fungi, several major plant pathogens and one oomycete were selected to clone the homologs, including *Puccinia striiformis* f. sp. *tritici*, *Pst* (causes wheat stripe rust), *Fusarium pseudograminearum*, *Fp* (causes wheat crown rot disease), *F. graminearum*, *Fg* (causes wheat head blight and crown rot), *Bipolaris sorokiniana*, *Bs* (causes wheat root rot), *Phytophthora infestans*, *Pi* (causes Solanaceae plants late blight), and *Verticillium dahliae*, *Vd* (cause cotton and Solanaceae plants Verticillium wilt). Interestingly, *BEC1019* homologs in biotrophic (*Pst*), hemi-biotrophic (*Pi*), and necrotrophic (*Ggt*, *Fp*, *Fg*, and *Vd*) pathogens are identical to those in *Bgh* (Accession No. AHZ59730.1) (Figure S1). The exception among the pathogens we examined was *Bs*, whose amino acid sequence showed 91.46% identity to that of *Bgh* (Figure S1).

### 2.2. BEC1019 Is Highly Expressed during Haustorium Formation by Bgt and Concomitant with the Development of Necrotic Symptoms in Ggt

**Figure 2:**
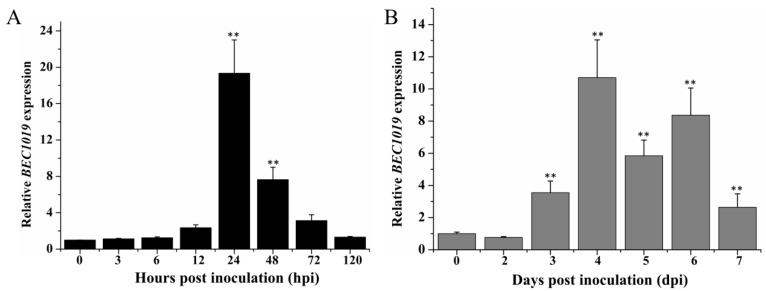
Relative expression patterns of *BEC1019* at different stages of *Blumeria graminis* f. sp. *tritici* (*Bgt*) and *Gaeumannomyces graminis* var. *tritici* (*Ggt*) infection. (**A**) Wheat leaves inoculated with *Bgt* were harvested at 0, 3, 6, 12, 24, 48, 72, and 120 h post-infection (hpi). (**B**) Wheat roots inoculated with *Ggt* were harvested at 0, 2, 3, 4, 5, 6, and 7 days post-infection (dpi). The *elongation factor 1α* (*ef-1α*) gene of *Bgt* and *18S rRNA* gene of *Ggt* were used as control genes for determinations of the relative expression of *BEC1019*. Bars indicate the means of three independent biological repetitions with standard errors. Double asterisks indicate significant differences relative to 0 hpi/dpi (*p* ≤ 0.01, according to *t*-tests).

To investigate whether *Bgt* and *Ggt* employ the effector gene *BEC1019* to facilitate host plant colonization and interfere with the different layers of wheat defenses induced during infection, we performed qRT-PCR to assess *BEC1019* expression in *Bgt*-infected wheat leaves at different time points (0, 3, 6 12, 24, 48, 72, and 120 hpi) and in *Ggt*-infected wheat roots at different stages (0, 2, 3, 4, 5, 6, and 7 dpi). With regards to *Bgt,* the aforementioned time points represent the following stages of infection: non-germinated conidia (0 hpi), primary germ tube formation (3 hpi), appressorial germ tube formation (6 hpi), penetration (12 hpi), haustorium formation (24 hpi), secondary penetration (48 hpi), microcolony formation (72 hpi), and conidiophore formation (120 hpi) [29,35]. Using the *elongation factor 1α* (*ef-1α*) gene of *Bgt* as an internal reference gene, we found that the transcript level of *BEC1019* was weakly induced during the first four stages of infection (0–12 hpi), whereas during haustorium formation (24 hpi), the abundance of *BEC1019* increased by 19-fold compared with that at 0 hpi. Thereafter, *BEC1019* expression gradually decreased with increasing *Bgt* colonization time, and at 120 hpi the *BEC1019* transcript level had declined to pre-inoculation levels (Figure 2A).

The time points at which we monitored *Ggt* infection represent the following stages: un-inoculated (0 dpi), hyphal infection of the root surface (2 dpi), small (1 mm) necrotic lesions observed (3 dpi), 3-mm necrotic lesions present and hyphae observed in the root cortex cells (4 dpi), 6-mm necrotic lesions present and alteration of host tissue observed (5 dpi), larger necrotic lesions observed (6 dpi), and vascular tissue completely colonized by the pathogen (7 dpi) [18]. Using the *18S rRNA* gene of *Ggt* as an internal reference gene, we observed that there was a marginal decrease in *BEC1019* expression during the early stage of *Ggt* colonization (2 dpi). However, concomitant with necrosis development, *BEC1019* was significantly up-regulated (3–6 dpi), peaking at 4 dpi. At this point, the root cortex cells had been colonized and *BEC1019* levels were 11-fold higher than those of the control. Thereafter, *BEC1019* transcript level showed a marginal decrease, concomitant with the complete colonization of vascular tissue (Figure 2B). Thus, our results indicate that *Bgt BEC1019* is involved in haustorium formation, whereas that of *Ggt* is associated with necrotic symptom development.

### 2.3. Overexpression of BEC1019 Increases Wheat Susceptibility to Bgt and Ggt, whereas Silencing BEC1019 Enhances Resistance to both Biotrophic and Necrotrophic Pathogens

**Figure 3:**
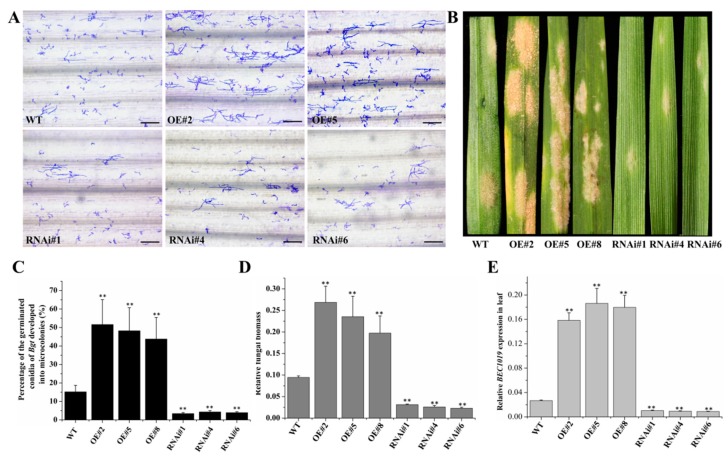
Overexpression of *BEC1019* in Aikang 58 wheat enhanced the susceptibility of plants to wheat powdery mildew *Blumeria graminis* f. sp. *tritici* (*Bgt*), whereas silencing *BEC1019* increased resistance to *Bgt*. (**A**) Microscopic analysis revealed *Bgt* microcolony formation on the leaves of control plants and *BEC1019*-overexpressing and RNAi plants at 60 h post-inoculation (hpi). Scale bar = 25 µm. (**B**) Macroscopic phenotypes of *Bgt* infection on wild-type control plant leaves and *BEC1019*-overexpressing and RNAi plant leaves. (**C**) Percentages of germinated *Bgt* conidia on the *BEC1019*-overexpressing and RNAi plants and control plants at 60 hpi. (**D**) Quantification of *Bgt* fungal biomass in control wheat and *BEC1019*-overexpressing and RNAi plants. (**E**) Transcript levels of *BEC1019* in *Bgt*-infected leaves of overexpressing and RNAi transgenic plants. Double asterisks indicate significant differences relative to control plants (*p* ≤ 0.01, according to *t*-tests).

To assess the roles of *BEC1019* in resistance development against wheat powdery mildew and take-all disease, transgenic plants harboring overexpression and RNAi vectors were inoculated with biotrophic (*Bgt*) and necrotrophic (*Ggt*) pathogens, respectively. The transgenic Aikang 58 wheat lines were initially inoculated with either water or fresh spores of *Bgt*, followed by microscopic observation at 60 hpi to calculate the percentages of germinated conidia that had developed into microcolonies. The results showed that the leaves of both overexpressing lines had a significantly larger number of *Bgt* microcolonies than did the leaves of control plants, whereas plants in which carrying *BEC1019* RNAi vector had fewer microcolonies (Figure 3A,C). We assessed macroscopic disease symptoms at 5–6 days after *Bgt* infection and found that symptoms observed in the leaves of the overexpressing and RNAi transgenic plants were consistent with the findings of our microscopic analyses (Figure 3B). Additionally, quantification of fungal biomass in *Bgt*-infected transgenic plants indicated significantly enhanced *Bgt* fungal biomass in *BEC1019*-overexpressing plants, whereas there was a considerable decrease in fungal biomass in *BEC1019*-silenced wheat lines (Figure 3D). Compared with the control plants inoculated with *Bgt*, the levels of *BEC1019/ef-1α* expression were considerably higher in the leaves of overexpressing lines and lower in the leaves of knockdown plants, as determined by qRT-PCR (Figure 3E).

**Figure 4:**
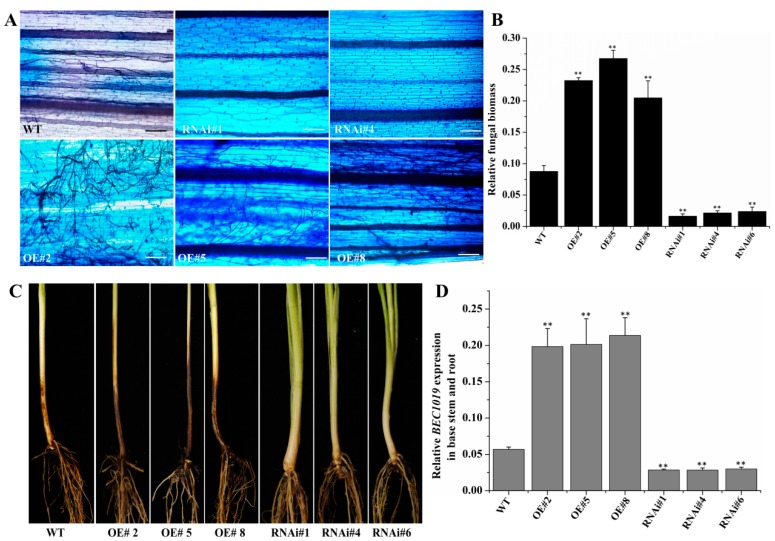
Overexpression of *BEC1019* in Zhoumai 26 wheat increased susceptibility to the necrotrophic pathogen *Gaeumannomyces graminis* var. *tritici* (*Ggt*), whereas silencing *BEC1019* decreased susceptibility to *Ggt*. (**A**) Macroscopic responses to *Ggt* infection on the stems and roots of *BEC1019*-overexpressing and RNAi lines and wild-type control plants. (**B**) Relative *Ggt* biomass of control wheat and *BEC1019* overexpressing and RNAi plants. (**C**) Microscopic analysis showing the growth of *Ggt* hyphae on the stems of wild-type plants and *BEC1019*-overexpressing and RNAi plants after inoculation. Scale bar = 25 µm. (**D**) Transcript levels of BEC1019 in *Ggt*-infected base stems and roots of overexpressing and RNAi transgenic plants. Double asterisks indicate significant differences relative to the control plants (*p* ≤ 0.01, according to *t*-tests).

Interestingly, in response to *Ggt* infection, *BEC1019*-overexpressing and RNAi-silenced Zhoumai 26 wheat lines at 21 dpi exhibited a similar phenotype to that of *Bgt*-infected plants, and our micro- and macroscopic analyses indicated that up-regulation of *BEC1019* significantly increased the susceptibility of wheat to *Ggt* compared with that of the wild-type control. In contrast, silencing *BEC1019* considerably reduced the susceptibility of these plants (Figure 4A,C). Moreover, the relative biomass of *Ggt* was also increased in wheat lines overexpressing *BEC1019* and reduced in *BEC1019* RNAi lines (Figure 4B). Additionally, the levels of *BEC1019/18S rRNA* transcription in the base stem and root of *Ggt*-inoculated transgenic plants were similar to those in leaves inoculated with *Bgt* (Figure 4D). Semi-qRT-PCR results also revealed the presence of the ORF and partial sequence of *BEC1019* in both overexpressing and RNAi transgenic lines (Figure S2).

**Figure 5:**
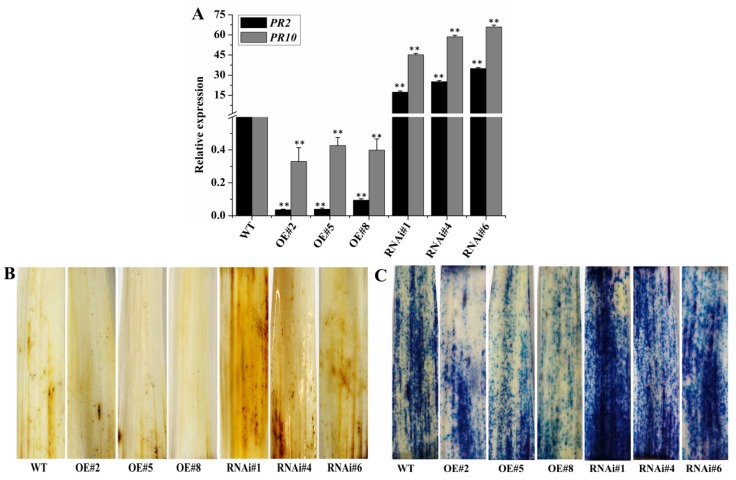
Defense-related traits identified in *BEC1019*-overexpressing and -silenced plants. (**A**) The results of qRT-PCR analyses showing the transcript levels of wheat defense marker genes *TaPR2* and *TaPR10* in *BEC1019*-overexpressing and RNAi plants. Double asterisks indicate significant differences relative to the control plants (*p* ≤ 0.01, according to *t* tests). (**B**) The leaves of wild-type wheat and *BEC1019*-overexpressing and RNAi plants were challenged with *Blumeria graminis* f. sp. *tritici* (*Bgt*) and sampled at 3 days post-infection (dpi) with DAB staining. (**C**) The leaves of wild-type wheat and *BEC1019* transgenic plants were challenged with *Bgt*, sampled at 1 dpi, and used to observe cell death, which was detected by staining with trypan blue.

Compared with the control plants, expression of the wheat *PR* marker genes *TaPR2* and *TaPR10* in *Bgt* non-inoculated transgenic lines was significantly down-regulated in *BEC1019*-overexpressing wheat lines and up-regulated in *BEC1019* RNAi lines (Figure 5A). Notably, in the silenced lines, the relative transcript levels of *TaPR2* and *TaPR10* were up-regulated by 17- to 66-fold compared with the controls (Figure 5A). To assess the effects of H_2_O_2_ accumulation and HR response in transgenic plants inoculated with *Bgt*, we performed DAB and trypan blue staining. At 3 dpi, DAB staining revealed a higher accumulation of H_2_O_2_ in *BEC1019* RNAi lines than in those overexpressing *BEC1019* and the controls (Figure 5B). Trypan blue analysis indicated a substantial increase in cell death in the *BEC1019* RNAi lines, whereas the cell death observed in *BEC1019*-overexpressing plants was comparable to that detected in the wild-type control, all of which were inoculated with *Bgt* and sampled a 1 dpi (Figure 5C). These findings were consistent with the microscopic and macroscopic observations for *Bgt*.

### 2.4. Transient Expression of BEC1019 Protein Triggers Cell Death in Nicotiana benthamiana

**Figure 6:**
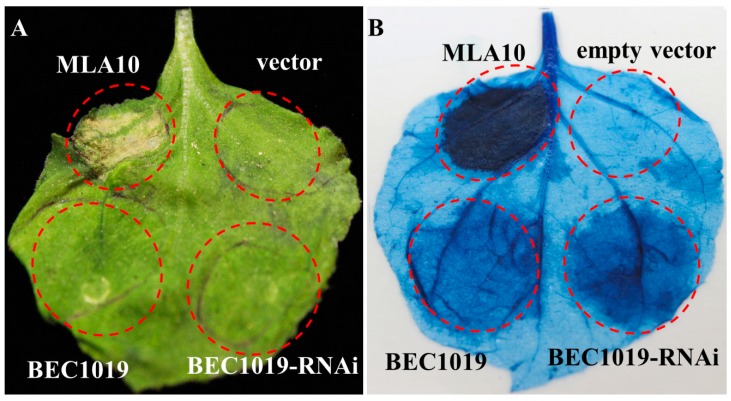
Analysis of cell death triggered by BEC1019 in *Nicotiana benthamiana*. The barley resistance gene *MLA10* (positive control) and full-length and RNAi partial sequences of *BEC1019* were expressed via agro-infiltration in *N. benthamiana* (**A**), and cell death representing HR was visualized by trypan blue staining (**B**) at 5 days post-agro-infiltration. No HR induction was detected in the negative control (empty vector).

To determine whether the full-length and RNAi partial sequence of *BEC1019* can trigger cell death, we performed *Agrobacterium tumefaciens*-mediated transient expression. The barley NLR immune receptor MLA10 was used as a positive control, the overexpression of which can induce a significant cell death phenotype in *N. benthamiana* leaves [36]. Accordingly, we observed that the transient expression of MLA10, CTAPi-GW-3HA-BEC1019, and CTAPi-GW-3HA-BEC1019-RNAi induced cell death (Figure 6), and MLA10 induced a typical cell death response at 5 days after agro-infiltration of the leaves. Cell death induced by the expression of full-length *BEC1019* and RNAi segment 5 days after agro-infiltration was observed to be less pronounced than that caused by MLA10, indicating that BEC1019 might interact with certain disease resistance proteins to trigger cell death in *N. benthamiana*.

## 3. Discussion

The sequencing and annotation of the genomes of *Bgh* and *Bgt* in 2010 and 2013, respectively [27,28], provided valuable new genetic resources for research on powdery mildews. However, owing to difficulties in obtaining transgenic wheat plants, most studies on the interaction of powdery mildews and plants have been performed using barley and *Bgh*. Numerous *Bgh*-specific *CSEPs* and *BECs* are considered candidate effector genes and are predicted to be expressed mainly in haustoria. HIGS technology has considerably enhanced the functional verification of fungal genes implicated in the interactions between plants and pathogens [31], particularly *Bgh* [29,30,31,32,37] and other biotrophic fungal pathogens, such as *Puccinia striiformis* f. sp. *tritici* [38] and *Fusarium graminearum* [39]. Previous studies in which *BEC1019* was silenced using HIGS have revealed the roles of this gene in *Bgh* colonization and haustorial development [33,34]. In the present study, we used HIGS to verify the roles played *BEC1019*, a conserved effector gene in *Bgh*, in *Bgt* and *Ggt*, which are, respectively, important obligate biotrophic and soil-borne necrotrophic fungi. The results showed that the *BEC1019* homologs in *Bgt* and *Ggt* are identical to those in *Bgh*, and similarly enhance the colonization of these pathogens in wheat, which was consistent with previous research that conserved effector genes played the similar roles in different bacterial pathogens [40,41].

Previous studies have also found that homologs of the *Bgh BEC1019* gene are present in the sequenced genomes of 96 other fungal pathogens [33,34]. Consistent with this finding, we identified *BEC1019* homologous sequences in eight pathogens of cereal crops and Solanaceae species, including biotrophic, hemi-biotrophic, and necrotrophic fungi, sharing 100% sequence identity with *Bgh BEC1019*. The only exception among the pathogens we examined was *Bipolaris sorokiniana*, which showed certain differences in sequences at the 5′ and 3′ ends of *BEC1019.* However, given that we found that the homolog in the oomycete *Phytophthora infestans* (hemi-biotrophy) has 100% sequence identity with *Bgh BEC1019*, we cannot conclude that hemi-biotrophic pathogens have specific variants of this gene. Thus, in the future, homologous sequences in other hemi-biotrophic pathogens should be examined to gain a better understanding in this respect. It is speculated that *BEC1019* might be an ancient gene that plays vital roles in the life cycles of fungi, and novel function, such as promoting the infection of plants, was acquired during the evolution of fungal pathogens.

Effector proteins are differentially expressed and assumed to be required at different stages of powdery mildew infection. Consistent with this notion, the *Bgh* effectors *CSEP0081*, *CSEP0254*, *CSEP0055*, *CSEP0105*, and *CSEP0162* were only detected at low levels during the early stages of infection (0–12 hpi) [29,30,42,43]. However, from 24 hpi (haustorium formation), there was a substantial increase in effector transcript levels, with some (*CSEP0105* and *CSEP0254*) showing peak expression at this stage, whereas the expression of other effectors (*CSEP0081*, *CSEP0055*, and *CSEP0162*) was further elevated at 48 hpi (secondary penetration). Our results indicated that *BEC1019* expression in *Bgt* is similar to that of *BEC1019*, *CSEP0105,* and *CSEP0254* in *Bgh*, with maximum expression coinciding with haustorium formation (24 hpi), thereby indicating that *BEC1019* is probably involved in haustorium formation in *Bgt*.

To date, a few effector genes have been isolated from the biotrophic fungi *Bgt*, the first of which, *AvrPm3^a2/f2^* (cloned in 2015), is abundantly expressed during haustorium formation and is specifically recognized by the wheat resistance genes *Pm3a* and *Pm3f* [43]. *AvrPm2* encodes an RNase-like effector protein that belongs to a structurally conserved gene family [44], which includes the *Bgh* effector gene *Avra13* (*CSEP0372*), and interacts with the barley resistance gene *MLA13* [26]. Two additional well-characterized RNase-like effector genes in *Bgh* are *BEC1011* [31] and *BEC1054* [45], and all the *Bgt* and *Bgh* RNase-like effectors genes have been found to have similar structural homologies. To evade plant immune systems, the effectors *AvrPm3^a2/f2^* and *AvrPm2* have evolved under strong diversifying selection [43,44], which contrasts with the broadly conserved *BEC1019*. However, no similar effector genes have been identified in *Ggt*, and accordingly, the majority of studies on resistance to *Ggt* have focused on the overexpression of heterologous proteins in wheat to enhance resistance to this pathogen. For example, the overexpression of soybean *GmPGIP3*, an antimicrobial peptide found in potato, and the *MYB* gene of *Thinopyrum intermedium* in wheat have been shown to confer increased resistance to take-all disease caused by the colonization of *Ggt* [46,47,48].

In the present study, we found that overexpression of *BEC1019* in wheat significantly promoted *Bgt* and *Ggt* virulence, thereby increasing the susceptibility of infected plants. Consistent with the role of *BEC1019* in the *Bgh* infection of barley, silencing of this gene considerably reduced *Bgt* colonization in wheat epidermal cells and *Ggt* infection in wheat stems and roots, which is related to HR and H_2_O_2_ accumulation. Therefore, we conclude that *BEC1019* is a highly conserved effector gene in fungi that is not only associated with biotrophic pathogen development but is also involved in necrotrophic fungal virulence. Accordingly, *BEC1019* may serve as a common candidate effector gene that can be used to identify the proteins with which it interacts, thereby providing an alternative strategy for elucidating the mechanisms underlying resistance to *BEC1019*. Furthermore, this strategy could contribute to the breeding of cereal crops with long-lasting broad-spectrum resistance.

## 4. Materials and Methods

### 4.1. Plants and Fungal Materials

Wheat (*Triticum aestivum* L.) Aikang 58 and Zhoumai 26, provided by the Henan Institute of Science and Technology and the Zhoukou Academy of Agricultural Sciences, respectively, were used as transformation recipients in this study. Cultivars of Aikang 58 and Zhoumai 26 were susceptible to *Bgt* and *Ggt*, respectively. The naturally occurring biotrophic fungus *B. graminis* f. sp. *tritici* (*Bgt*) was isolated from material collected in a field in Zhoukou, Henan Province, China (33°62ʹN, 114°65ʹE), and maintained on Zhoumai 22, grown under a 16-h light (22 °C) and 8-h dark (20 °C) photoperiod with 60% atmospheric humidity.

*Ggt* LY3-21, the wheat hemi-biotroph *Bipolaris sorokiniana* (*Bs*), and the necrotrophic pathogens *Fusarium pseudograminearum* (*Fp*) and *F. graminearum* (*Fg*) were provided by the Henan Academy of Agricultural Science and cultured on potato dextrose agar at 22–25 °C. *Puccinia striiformis* f. sp. *tritici* (*Pst*), a gift from the Zhoukou Academy of Agricultural Sciences, was cultured on the susceptible wheat cultivar Mingxian 169 in a growth chamber under a 16-h light (16 °C) and 8-h dark (8 °C) photoperiod. The Solanaceae pathogens *Phytophthora infestans* (*Pi*) and *Verticillium dahliae* (*Vd*), provided by the Chinese Academy of Agricultural Sciences, were cultured on rye agar medium and Czapek–Dox medium, respectively.

### 4.2. Cloning of BEC1019 Homologous Genes

For DNA extraction, four to five leaves of *Bgt*-infected 7-day-old wheat, *Pst*-infected 12-day-old wheat and the other fungi cultivated on medium were ground to a powder in liquid nitrogen using a precooled mortar and pestle, according to the manufacturer’s instructions (SK8230; Shanghai Sangon Biological Engineering Corporation, Shanghai, China) of DNA extraction kit. The *BEC1019* gene homologs were amplified using the BEC1019-F/R primer pair, designed based on the *Bgh BEC1019* sequence. The polymerase chain reaction (PCR) program used for amplification was as follows: pre-denaturation at 94 °C for 5 min; followed by 30 cycles at 94 °C for 30 s, 58 °C for 45 s, and 72 °C for 1 min; with a final extension at 72 °C for 10 min. Corresponding amplicons were inserted into a pEASY-Blunt cloning vector (Beijing TransGen Biotech Corporation, Beijing, China) and recombinant plasmids were sequenced using the universal M13 primer.

### 4.3. Construction of BEC1019 Overexpression and RNAi Vectors

The *BEC1019* sequence containing the full-length open reading frame (ORF) was amplified from *Bgt* DNA using the primer pair BEC1019-F1/R1, designed based on the *BEC1019* sequence (GenBank accession no. KJ571201). To clone a 264-bp region (368–632 bp) of *BEC1019*, we designed the primer pair BEC1019-F2/R2 based on the sequence used in Barley stripe mosaic virus-induced gene silencing of *Bgh* [33,34], Gateway technology was used to construct overexpression and RNAi vectors. Initially, we inserted the complete and RNAi partial sequences of *BEC1019* into a pDONR207 intermediate vector, generating pDONR-BEC1019 and pDONR-*BEC1019*-RNAi vectors, respectively. Recombining these into gateway destination vectors, CTAPi-GW-3HA and modified pCAMBIA2301 using LR clonase, generated the final vectors [49]. The primers used for amplification are listed in Table 1. The CTAPi-GW-3HA-*BEC1019* and pCAMBIA2301-*BEC1019*-RNAi vectors were used to generate transgenic wheat lines, and *BEC1019* transgenic T_1_ wheat was provided by the Plant Genetic Transformation Center of Henan Key Laboratory of Crop Molecular Breeding and Bioreactor. The CTAPi-GW-3HA-*BEC1019* and CTAPi-GW-3HA-*BEC1019*-RNAi vectors were used to transiently transform in *Nicotiana benthamiana* leaves for the detection of cell death.

### 4.4. DNA and RNA Extraction and Quantitative Real-Time PCR

To evaluate the transcript levels of *BEC1019* produced in response to *Bgt* and *Ggt* infection, wheat Aikang 58 leaves were sampled at 0, 3, 6, 12, 24, 48, 72, and 120 h post-infection (hpi) with *Bgt* and wheat Zhoumai 22 base stems and roots at 0, 2, 3, 4, 5, 6, and 7 days post-infection (dpi) with *Ggt*. To quantify fungal biomass, we used *Bgt* and *Ggt* DNA extracted from inoculated wheat leaves, using a DNeasy plant mini kit (QIAGEN, Dusseldorf, Germany). Total RNA used for the quantification of transcript levels was extracted from wheat leaves using TRIzol reagent (Life Technologies, Grand Island, NY, USA) according to the manufacturer’s instructions. The extracted RNA was treated with RNase-free DNase I (Takara Bio Inc., Shiga, Japan) to remove contaminant genomic DNA. First-strand complementary DNA (cDNA) was synthesized using a PrimeScript RT Perfect Real Time reagent kit (Takara Bio Inc., Shiga, Japan). The synthesized cDNAs were used as templates in the following PCRs. Quantitative reverse transcription PCR (qRT-PCR) was performed using the CFX96™ real-time PCR detection system (Bio-Rad, Hercules, CA, USA) with SYBR^®^ Premix Ex Taq™ (Tli RNaseH Plus; Takara Bio Inc., Shiga, Japan). The PCR program used was as follows: 95 °C for 3 min, followed by 40 cycles at 95 °C for 10 s and 60 °C for 30 s. The *ef-1α* gene of *Bgt* (HM538432) and *18S rRNA* gene of *Ggt* (FJ771002) were used for fungal quantification [50,51], and the *TaActin* (KC775780) gene was used as the wheat internal reference for normalization. The transcript levels of *BEC1019* and the other genes were examined and fungal biomass were calculated using the 2^−ΔΔC*t*^ method [52]. All assays for a particular gene were performed synchronously in triplicate under identical conditions, and all reactions had three biological replicates. The sequences of the primers used for the qRT-PCR are listed in Table 1.

### 4.5. Detection of H_2_O_2_ and Cell Death

H_2_O_2_ was detected histochemically using the 3,3-diaminobenzidine (DAB; Aladdin, Shanghai, China) staining method according to Thordal-Christensen et al. [53]. Leaves at 3 dpi with *Bgt* were stained with 1 mg/mL DAB dissolved in HCl-acidified (pH 3.8) distilled water for 6–8 h. Chlorophyll was extracted overnight in acetic acid:ethanol solution (3:1), and the leaves were stored in 50% glycerol. Leaves at 24 hpi with *Bgt* were stained with 0.4% trypan blue solution at 90 °C for 1 min. Chlorophyll was extracted in acetic acid:ethanol solution (3:1) at 37 °C, and the leaves were stored in 50% glycerol for subsequent observation [36].

*N. benthamiana* leaves were used to visualize cell death using the trypan blue staining method [36]. The CTAPi-GW-3HA-BEC1019 and CTAPi-GW-3HA-BEC1019-RNAi vectors were transformed into *A. tumefaciens* GV3101, and on reaching an OD_600_ of 0.2–0.5, the *A. tumefaciens* GV3101 cells carrying either of the two aforementioned vectors were infiltrated into *N. benthamiana* leaves. The leaves were sampled at 5 dpi and boiled for 5 min in trypan blue staining solution (20 mL lactic acid, 20 mL glycerol, 20 g phenol, and 20 mg trypan blue, dissolved in 20 mL distilled water), and subsequently de-stained in 2.5 g/mL chloral hydrate for 2–3 days.

### 4.6. Responses of Transgenic Wheat Plants to Bgt and Ggt

Overexpressing and RNAi transgenic Aikang 58 wheat leaves were inoculated with *Bgt* and the subsequently infected leaves were microscopically analyzed using previously described procedures [24], with minor modifications. Leaf segments were collected from three 1-week-old transgenic wheat lines and placed on a 1% agar plate containing 85 µM benzimidazole. These were incubated in a 20 °C climate chamber for more than 4 h and then inoculated with *Bgt* spores by gently spreading fresh conidia on the leaves at an appropriate density (~250 conidiospores per·cm^2^ of leaf). *Bgt* was allowed to develop on the leaves for 60 h, and then the leaf segments were fixed in ethanol/acetic acid (1:1, *v*/*v*), stained with Coomassie brilliant blue R250 in methanol (0.6%, *w*/*v*) for 10 s, rinsed in deionized water, and observed and enumerated under a BX61 upright microscope (Olympus, Tokyo, Japan). More than 1000 germinated spores on each leaf segment from each plant were observed. The phenotypes of the inoculated transgenic leaves were observed and photographed using a Canon EOS 600D camera (Canon Corp., Tokyo, Japan) at 7 dpi.

*Ggt* inoculation was based on the method described by Liu et al. [46]. Fresh plugs (4 × 4 cm^2^) containing *Ggt* colonies were placed on the surface of sterilized soil, and then 1-week-old seedlings of transgenic Zhoumai 26 were placed on top of the *Ggt* plugs and covered with 3 cm of soil. The plants were cultured in a climate box under a 16-h light (22 °C)/8-h dark (16 °C) regime at 60% relative humidity. For microscopic observations of *Ggt* fungal growth, the base sheaths of the seedlings were harvested at 21 dpi, fixed with 1:1 (*v*/*v*) ethanol/acetic acid for 24 h, stained with trypan blue for 6 h, and examined under a BX61 microscope.

### 4.7. Data Analysis

Data were statistically analyzed using an independent-sample *t*-test and one-way analysis of variance (ANOVA) followed by post hoc comparisons using the least significant difference (LSD) and Duncan tests (*p* ≤ 0.01). All analyses were performed using the statistical package for the social sciences (SPSS) version 17 following the instructions in the Survival Manual.

## Figures and Tables

**Table 1:** Primers names and sequences used in this study.

Primer Name	Primer Sequence (5′-3′)	Primers Purpose
BEC1019-F	ATGCAGTCTGTATTGCTTTT	Homolog gene cloning of *BEC1019*
BEC1019-R	CTAGACACAATGAACCTCGC	
BEC1019-F1	GGGGACAGTTTGTACAAAAAAGCAGGCTTCATGCAGTCTGTATTGCTTTT	Construction of CTAPi-GW-3HA-*BEC1019* vector
BEC1019-R1	GGGGACCACTTTGTACAAGAAAGCTGGGTGACACAATGAACCTCGCCAT	
BEC1019-F2	GGGGACAGTTTGTACAAAAAAGCAGGCTTCGTGATGACCCGGACAAAA	Construction of pCAMBIA2301-*BEC1019*-RNAi vector
BEC1019-R2	GGGGACCACTTTGTACAAGAAAGCTGGGTAGGGCATCTTGGTAACCA	
BEC1019-F3	GGGGACAGTTTGTACAAAAAAGCAGGCTTCGTGATGACCCGGACAAAA	Construction of CTAPi-GW-3HA-*BEC1019*-RNAi vector
BEC1019-R3	GGGGACCACTTTGTACAAGAAAGCTGGGTAGGGCATCTTGGTAACCA	
BEC1019-F4	TCATGTGGACATCGTCGGTC	q RT-PCR analysis of *BEC1019* over-expression wheat lines
BEC1019-R4	CACGCTGATGTCAAACGCAT	
BEC1019-F5	AATGTGCAACCGAGAACCGA	q RT-PCR analysis of *BEC1019* silencing wheat lines
BEC1019-R5	TCCTCCAAAGGAAGCCGTTC	
TaPR2-F	CCGGCCATACTACCCGGC	q RT-PCR analysis for *TaPR2*
TaPR2-R	ACACCTTGATGGCGCTGAGA	
TaPR10-F	ACGGAGCGGATGTGGAAG	q RT-PCR analysis for *TaPR10*
TaPR10-R	GCCACCTGCGACTTGAGC	
TaActin-F	CCAGGTATCGCTGACCGTAT	Reference gene of wheat
TaActin-R	GCTGAGTGAGGCTAGGATGG	
Bgt-EF1a-F	GTCGGATTTAACCCCAAGGT	Reference gene of *Bgt*
Bgt-EF1a-R	TTTATCGGTAGGGCGACTTG	
Ggt-18S rRNA-F	CGAACTCGGTCGTTTAGAGG	Reference gene of *Ggt*
Ggt-18S rRNA-R	GGTATGTTCACAGGGGTTGG	

## Supplementary Materials

Supplementary materials can be found at https://www.mdpi.com/1422-0067/20/18/4376/s1.
