# Supplementary material for: The Highly Conserved Barley Powdery Mildew Effector BEC1019 Confers Susceptibility to Biotrophic and Necrotrophic Pathogens in Wheat
Source: Int J Mol Sci. 2019 Sep 6;20(18):4376. doi: 10.3390/ijms20184376 (PMC6770355; doi:10.3390/ijms20184376)
Supplement: Supplementary file 1 [file ijms-20-04376-s001.pdf]

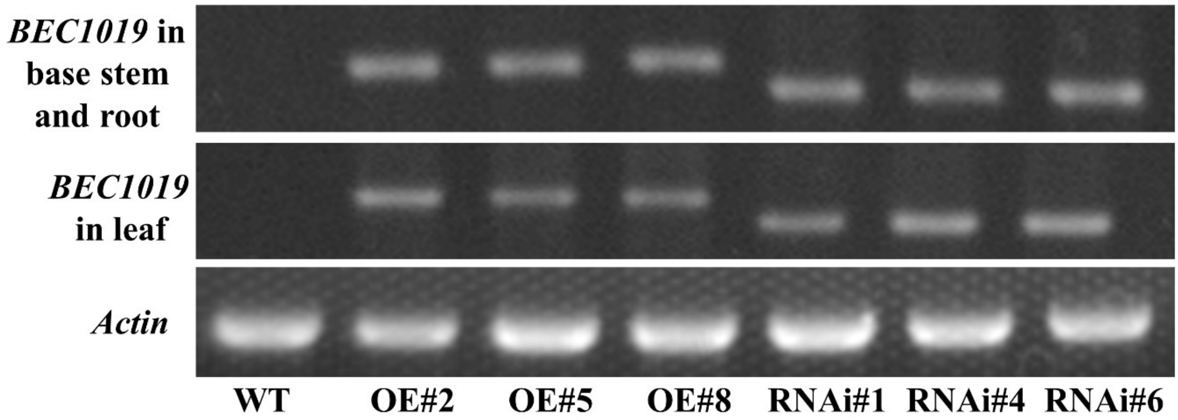

Figure 1. Amino acid sequence alignment of BEC1019 homologs in different plant pathogens using DNAMAN software.

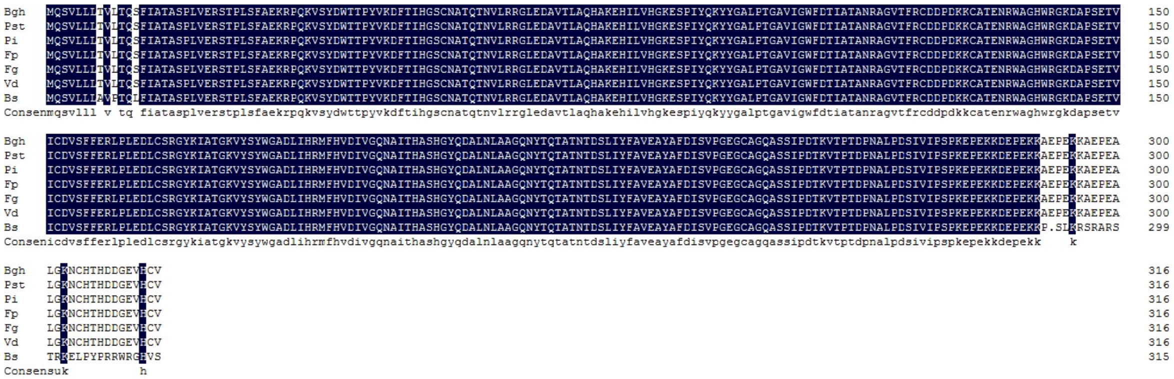

Figure 2. Semi-quantitative RT-PCR analysis of *BEC1019* expression in RNAi and overexpressing plants. *TaActin* transcripts were quantified as an internal control.
